# Scar among Bacillus Calmette-Guerin Vaccinated Children Presenting to an Immunisation Clinic at a Tertiary Hospital in Nepal: An Observational Study

**DOI:** 10.31729/jnma.9025

**Published:** 2025-05-31

**Authors:** Ajaya Kumar Dhakal, Divya KC, Barsha Shrestha, Devendra Shrestha, Nischal Neupane, Sharda Acharya, Saurav Shrestha

**Affiliations:** 1 Department of Paediatrics, KIST Medical College and Teaching Hospital, Imadol, Lalitpur, Nepal

**Keywords:** *BCG scar*, *BCG vaccination*, *Nepal*, *scar failure*

## Abstract

**Introduction:**

Bacillus Calmette-Guerin vaccination is associated with the development of scarring at the vaccination site in most children within 12 weeks of vaccination. However, due to various factors, some children do not develop scars. The aim of the study was to determine the prevalence of scar failure and the differences in clinical characteristics between scar-positive and scar-negative children.

**Methods:**

This observational cross-section study was conducted between 12 September 2022 and 15 April 2024 on children attending a vaccination clinic at a tertiary care hospital for 12 or 15-month vaccination. A purposive sampling method was used, and 386 children were enrolled in the study. During medical history, information was collected on Bacillus Clamettte-Guerin vaccination, child and maternal clinical profiles, and hospitalisations for respiratory tract infections in children.

**Results:**

Among 386 infants, prevalence of BCG scar was 341 (88.34%; 95% CI: 91.81 %-91.37%). Vaccination was confirmed in 94.82% by history and card. The median scar size was 4mm (IQR: 2,5 mm) transversely and 3 mm (IQR: 2,5) vertically. Thirty-two (8.30%) infants required hospital admission for respiratory infections; none received anti-tubercular treatment.

**Conclusions:**

The BCG scar formation was higher compare to the scar failure. The scar failure was comparable to other studies.

## INTRODUCTION

The presence of a Bacillus Calmette-Guerin (BCG) scar is a confirmatory marker for BCG vaccination.^1^ The vaccination is widely recommended due to its protective effect against disseminated tuberculous meningitis.^2^ There has been renewed interest in BCG scars because they are associated with reduced morbidity and mortality and improved survival rates in children and infants.^1,3,4^

Studies conducted in different geographical locations have shown different scar incidence rates in BCG-vaccinated children.^5-7^ The main reason for the failure of BCG scar formation in most studies was an improper BCG injection technique^8^ , type of BCG strain^8^, vaccine dose^9^, wheal size after vaccination^10^ and genetic factors. The vaccination coverage rate of the BCG vaccine in Nepal was 97.8% in 2021^11^; however, there is limited information on the prevalence of BCG scar in Nepalese children. Therefore, this study aimed to determine the prevalence of BCG scar in children attending a vaccination clinic at a tertiary hospital in Lalitpur, Nepal.

## METHODS

This hospital-based observational cross-section study was conducted on children attending a paediatric immunisation clinic at KIST Medical College and Teaching Hospital which is a tertiary medical centre in Lalitpur, Nepal. The study was conducted between 12 September 2022 and 15 April 2024 after approval from the Institutional Review Committee (Reference number: 2079/80/61). Children who visited the immunisation clinic for their 12 or 15-month vaccination were eligible to participate in the study. Guardians were informed of the purpose of the study before data collection, and written consent was obtained from all participants. The sample size was calculated using the following formula:

n = Z × p × q /e^2^

where, n = minimum required sample size

Z = 1.96 at 95% confidence interval (CI)

p = prevalence was taken as 50% to obtain the maximum sample size

q = 1-p

e = margin of error, 5%

The sample size of 386 was considered for this study.

All parents or relatives who attended the vaccination clinic were asked about their children's BCG vaccination timing as part of their medical history. The timing was also confirmed using the vaccination card or the hospital vaccination record when the vaccination card was unavailable. Vaccination locations such as hospitals, ward clinics, health posts, and primary health centres were also recorded. Maternal and birth details were recorded as part of the patient's medical history or from medical records. When the father or grandparents brought the child with them, and medical information about the mother was insufficient, the mother was contacted by telephone to obtain or clarify the information. Infants delivered before 37 completed weeks of gestation after the last menstrual cycle were classified as pre-term; those who delivered between 37 and 42 weeks of pregnancy were considered full-term, while those who delivered after 42 weeks of pregnancy were considered post-term. Pregnancy-related problems, such as gestational diabetes, preeclampsia, or any other problem requiring attention during pregnancy, were defined as antenatal complications. Exclusive breastfeeding was defined as an infant receiving only breast milk. Exclusive breastfeeding at one, three, and six months were assessed. In addition, hospitalisation for respiratory infections in children was also reviewed. The mother was identified as atopic based on an earlier diagnosis of atopic disease or a history suggestive of atopic disease symptoms.

The BCG scar was examined and, if present, measured with a rigid, transparent plastic ruler to determine the maximum length of the scar transversely (transverse length) and vertically along the long axis of the arm (vertical length). The scar was also examined for keloid formation. Children with multiple vaccination visits were enrolled only once. Patients with incomplete vaccination records and maternal details were excluded from the study. The collected data were processed using MS Excel and exported to SPSS Statistics for Windows version 16 (SPSS Inc., Chicago, III., USA) for further analysis. Prevalence of BCG scaring was estimated at 95% confidence interval (CI).

## RESULTS

This study included 386 infants who received the BCG vaccination. The prevalence of BCG scar was 341 (88.34%; 95% CI: 91.81%-91.37%). The BCG scar was present in 184 (53.96%) males, 313 (91.74%) term, 181 (53.08%) caesarean delivery, 333 (95.79%) singleton foetus, 293 (85.92%) newborn with birthweight 2.5-3.99 Kg, 284 (83.29%) neonate not requiring hospitalization and 295 (86.51%) children not requiring antiboitics in first three months of life (Table 1).

Vaccination history was collected from mothers 264 (68.40%), parents 92 (23.83%), fathers 25 (6.48%), and grandparents or other family members 5 (1.29%). The BCG vaccination was confirmed by history and vaccination card in 366 (94.82%) children, whereas in 20 (5.18%) it was confirmed by history and hospital vaccination record. The median transverse length of the scar was 4 mm (IQR: 2.5mm) while the median vertical length was 3 mm (IQR: 2,5mm). Of the patients who developed scars, 3 (0.088%) showed keloid formation at the vaccination site.

The mean maternal age at delivery in BCG scar-positive and BCG scar-negative children was 28.27±4.74 years and 28.49±5.36 years, respectively. The mean birth weight of the BCG scar-positive children was 2983.87±485.12 gm, and that of the BCG scar-negative children was 2860.56±490.52 gm. On evaluation of maternal characterstics, 295 (86.51%) child with exclusive breast feeding at first month of age, 298 (87.39%) child with mother at age 20-34 years and 297 (87.09%) child with mother not having antenatal complications had BCG scar. Similarly, 265 (77.71%) mother with BCG scar, 272 (79.77%) mother who did not smoke, 326 (95.60%) mother having no history of atopy and 301 (88.27%) mothers not having children less than four years at home had BCG scar positive (Table 2).

There were 193 (56.60%) children receiving BCG vaccine withing 72 hour (Table 3), the BCG vaccine, 99 (25.65%) parents reported swelling or ulceration at the vaccination site, while 287 (74.35%) either did not recall or did not notice swelling. There were 341 (88.34%) children with positive BCG scar having vaccination at hospital. Of the 386 infants, 32 (8.30%) required hospital admission for acute respiratory tract infection. Of the admitted children, 30 (93.75%) were BCG-scar-positive, and 2 (6.25%) had no BCG-scar. None of the children in the study received anti-tubercular medication during the study period (Table 3).

**Table 1:** Clinical characteristics of BCG scar-positive and scar-negative children (n=386).

		BCG scar-positive (n1=341) n (%)	BCG scar-negative (n2=45) n (%)
Sex	Male	184 (53.96)	23 (51.11)
	Female	157 (46.04)	22 (48.89)
Gestational age	Pre-term	26 (7.62)	4 (8.89)
	Term	313 (91.79)	41 (91.11)
	Post-term	2 (0.59)	0 (0)
Mode of delivery	Caesarean delivery	181 (53.08)	19 (42.22)
	Vaginal delivery	160 (46.92)	26 (57.78)
Number of foetuses	Singleton	333 (97.65)	45 (100)
	Multiple	8 (2.35)	0 (0.00)
Birthweight	<2.5 Kg	44 (12.91)	10 (22.22)
	2.5-3.99 Kg	293 (85.92)	35 (77.78)
	4 Kg	4 (1.17)	0 (0)
Neonatal problems requiring hospitalisation	Yes	57 (16.71)	8 (17.78)
	No	284 (83.29)	37 (82.22)
Antibiotics in the first three months of life	Yes	46 (13.49)	6 (13.33)
	No	295(86.51)	39(86.67)

**Table 2:** Maternal characteristics of BCG scar-positive and scar-negative children (n=386).

		BCG scar-positive (n1=341) n (%)	BCG scar-negative (n2=45) n (%)
Feeding at the first month of age	Exclusive breastfeeding	295 (86.51	39 (86.67)
	Non-exclusive breastfeeding	46 (13.49)	6 (13.33)
Feeding in the first three months of age	Exclusive breastfeeding	274 (80.35)	38 (84.44)
	Non-exclusive breastfeeding	67 (19.65)	7 (15.56)
Feeding in the first six months of age	Exclusive breastfeeding	137 (40.18)	20 (44.44)
	Non-exclusive breastfeeding	204 (59.82)	25 (55.56)
Maternal age	<20 years	9 (2.64)	3 (6.67)
	20-34 years	298 (87.39)	36 (80.00)
	35 years	34 (9.97)	6 (13.33)
Antenatal complications	Yes	44 (12.91)	4 (8.89)
	No	297 (87.09)	41 (91.11)
Maternal BCG scar	Yes	265 (77.71)	28 (62.22)
	No	76 (22.29)	17 (37.78)
Parental smoking	Yes	69 (20.23)	8 (17.78)
	No	272 (79.77)	37 (82.22)
Maternal atopic history	Yes	15 (4.40)	0 (0)
	No	326 (95.60)	45 (100)
Child < 4 years at home	Yes	40 (11.73)	5 (11.11)
	No	301 (88.27)	40 (88.89)

**Table 3:** Comparison of vaccination timing after birth and vaccination location between BCG scar-positive and scar-negative children (n=386).

		BCG scar-positive (n1=341) n(%)	BCG scar-negative (n2=45) n(%)
Vaccination timing after birth	Birth-72 hours	193(56.60)	28(62.22)
	73 hours-7 days	67(19.65)	9(20.00)
	8-14 days	36(10.56)	7(15.56)
	15-21 days	21(6.16)	0(0)
	22-28 days	4(1.17)	0(0)
	>29 days	20(5.87)	1(2.22)
Vaccination location	Hospital	321(94.12)	42(93.33)
	Other health centres	20(5.88)	3(6.67)

## DISCUSSION

This study showed that the scar failure rate of BCG vaccination in the study population was 11.66%. The scar failure rate of BCG in the present study was comparable to a study conducted in Pakistan (11%).^5^ Other studies from India and Denmark reported a similar scar failure rate of 9%.^7,9^ Furthermore, the literature review revealed a wide variation in scar failure rates, ranging from 2% to 48% in studies from different geographical locations.^4,6,8^ The mean scar size in this study was similar to that of previous studies^7^, and one study found that the larger the scar, the greater the experience of the vaccinator.^9^ BCG scarring was associated with the development of a positive tuberculin skin test, and the size of the tuberculin skin test was directly proportional to the scar size in the previous study.^7^ The factors that predict scar formation after vaccination are post-vaccination wheal^10^, a good BCG injection technique, years of experience with vaccinators, and vaccination month.^9^

The male population was marginally more in both BCG scar positive and negative group, 184 (53.89%) and 23 (51.11%) similarly proportion of term delivery were more in both group, suggesting no direct relationship of gender and gestational age to BCG scar. Similar observation was found in other studies as well.^5,9,^ However, one study that revealed that post-term had the highest scar failure (22.2%) compared with term (3.2%) and pre-term (0%).^6^ In this study BCG scar failure was observed more in vaginal delivery 26 (57.78%) however, in one of the study mode of delivery did not affect BCG scarring.^9^ This study did not observed any specific pattern in relation to birthweight, however, the postive and negative BCG scarring was almost equal in neonate requiring hospitalization and those wo received antibiotics in the first three month of life as per the descriptive analysis.

The type of feeding, whether exclusive or nonexclusive breastfeeding, at one month, three months, and six months, was similar between scar-positive and scar-negative children in this and prior studies.^5^

Maternal age, parental smoking status, and having children less than four years did not show any specific variation in those who were BCG scar positive and negative. These findings are consistent with those of previous studies.^9^ Previous studies have shown that atopic disposition in parents increases the risk of scar failure^9^, however in our study we had 15 mothers with the history of atopy and non of the child from those mother were BCG scar negative. The number of the mother with history of atopy is not sufficient to make any conclusion in our study. In mothers who did not have BCG scars, 76 (22.29%). In one of the study association between a maternal scar and an infant scar was found^9^ suggesting that parental genetics may influence BCG scar formation in children. In addition, studies have shown that parental BCG vaccine scars decrease infant mortality in the first six weeks of life.^12^

Most of the children were vaccinated within 72 hour of birth. The timing of BCG vaccination after birth did not affect the formation of the BCG scar in previous studies.^5,9^ The frequency of hospitalisation for acute respiratory infection was similar in scar-positive and scar-negative children. However, previous studies have highlighted the protective effect of BCG scars against pneumonia and bronchial asthma during childhood.^13^ None of the infants were diagnosed with tuberculosis or took anti-tubercular medication during the study period. Some infants in this study developed vaccination site keloid, and previous studies have also shown that BCG-induced keloid is one cause of keloid, particularly in women.^14^

There are no established guidelines for the treatment of BCG scar-negative children.^7^ The Indian Academy of Pediatrics guidelines on immunisation recommend revaccination with a second dose of BCG vaccine if the scar is not visible six months after vaccination. Other authors consider BCG revaccination in children to be unnecessary. Given current evidence that the injection of a second dose of BCG has minimal systemic adverse effects and provides several non-specific benefits in addition to protection against tuberculosis, children without BCG scars can benefit from BCG revaccination.^2,15^

One of the significant limitations of this study was the small number of post-term infants and macrosomic babies, which made it difficult to generalise this study to this population. Although the maternal scar was assessed, the paternal BCG scar, which could have influenced scar formation due to genetic predisposition, was not examined. In addition, this study did not examine the BCG injection technique and vaccinator experience, which are significant factors in scar formation.

## CONCLUSION

This study found a BCG scar was observed in most of the children. The failure rate was comparable to other studies. Scar formation showed little variation across gender, gestational age, birthweight, feeding type, and maternal factors such as age or smoking. Children of mothers without a BCG scar were more frequently scar-negative. Most infants received vaccination within 72 hours of birth. The occurrence of acute respiratory infection was comparable between scar-positive and scar-negative children.
